# The Persistent Challenge of Advanced HIV Disease and AIDS in the Era of Antiretroviral Therapy

**DOI:** 10.1093/cid/cix1138

**Published:** 2018-03-04

**Authors:** Alexandra Calmy, Nathan Ford, Graeme Meintjes

**Affiliations:** 1 Division of Infectious Diseases, HIV Unit, Department of Internal Medicine Specialties, Geneva University Hospitals and Faculty of Medicine; 2 Department of HIV, World Health Organization, Geneva, Switzerland; 3 Wellcome Trust Centre for Infectious Diseases Research in Africa, Institute of Infectious Disease and Molecular Medicine; 4 Department of Medicine, Faculty of Health Sciences, University of Cape Town, South Africa

**Keywords:** advanced HIV disease, AIDS, cryptococcal meningitis, tuberculosis

There has been tremendous progress in improving access to antiretroviral therapy (ART) over the past decade, supported by major scientific advances, national and international political and financial commitment, and civil society engagement. Overall global efforts to deliver ART at scale have been extremely successful in reducing mortality, and governments are committed to ending AIDS as a public health threat by 2030. Nevertheless, there is an increasing realization that there is a persistent and large burden of human immunodeficiency virus (HIV)–associated morbidity and mortality encountered in countries most affected by HIV. A substantial proportion of patients is still at risk of death due to progressing to advanced HIV—defined by the World Health Organization (WHO) as having a CD4 count <200 cells/µL or WHO clinical stage 3 or 4 disease—and this proportion has remained relatively constant in recent years despite ongoing improvements in access to ART (Figure 1).

**Figure 1.**
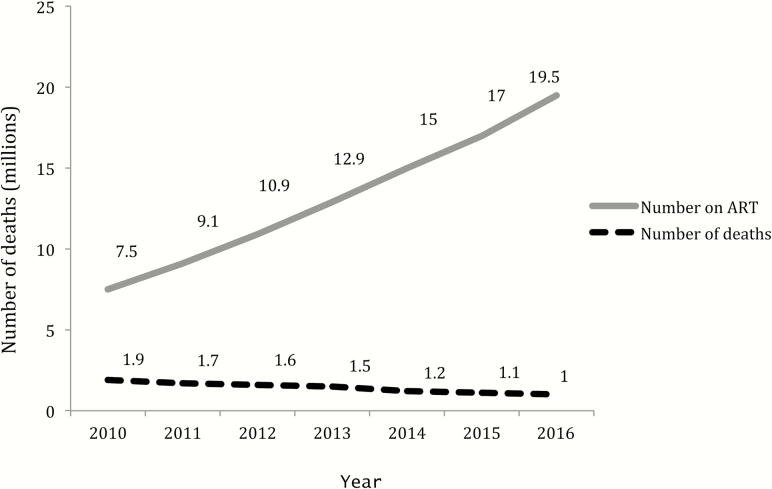
Number of patients receiving ART and number of deaths. Abbreviation: ART, antiretroviral therapy.

This supplement brings together a number of articles from a diverse group of authors that highlight the ongoing burden of advanced HIV disease, and opportunities to improve treatment and care for this highly vulnerable patient population. The studies reported in this supplement were used to inform the development of World Health Organization guidelines for the management of advanced HIV disease. The issues and recommendations discussed in the supplement largely pertain to lower- and middle-income countries (LMICs). We recognize that the opportunistic disease burden encountered in LMICs is in many ways different to that encountered in high-income countries and healthcare resources are limited, necessitating a public health approach for the management of late presenters and patients with AIDS.

The enduring burden of advanced HIV disease is described in 3 original research articles in this supplement [1–3].

Two studies from South Africa, using different data sources, both report that a substantial proportion of HIV-infected individuals starting ART did so with advanced HIV disease. The first study, using nationwide laboratory data to determine first CD4 cell count at entry into care, found that of 654868 patients entering care in 2016, almost a third (32.9%) had advanced HIV disease; men were almost twice as likely as women to enter care with very advanced HIV disease [1]. The second study, using data from a province-wide cohort of all HIV patients receiving CD4 count testing in the public sector in South Africa, found that the proportion of people with very advanced disease (defined as CD4 count <50 cells/µL) is not declining despite the successful scaling up of ART [2]. Late ART initiation is a worldwide problem. A recent analysis of data from HIV programs across 55 countries has found that the proportion of patients presenting to care with CD4 counts <200, <100, and <50 cells/µL has plateaued since 2010 [4]. Even in Switzerland, where ART coverage is high and universally available, up to 25% of people starting ART do so at a CD4 cell count <200 cells/µL (Alexandra Scherrer, personal communication).

The second study from South Africa further highlights a relatively new understanding, that an increasing proportion of people with very advanced HIV disease are ART experienced, and in this study increased from 14.3% in 2008 to 56.7% in 2017 [2]. Again, men were at greater risk [2], a finding reported by other studies [4, 5]. These findings are supported by a study from Kenya and the Democratic Republic of Congo (DRC) using data from inpatient departments in Médecins Sans Frontières–supported hospital wards [3], which found that the majority of HIV-infected hospitalized patients in both sites were ART experienced. Further research is needed to inform the management of this group of patients, including approaches to encourage and welcome those who have disengaged to return to care.

Taken together, these articles add to a growing evidence base showing that advanced HIV disease remains a persistent challenge globally [4–6].

Causes and timing of mortality and morbidity among patients with advanced HIV disease are addressed by 3 articles in the supplement [3, 7, 8].

A report from the REALITY randomized trial conducted in Zimbabwe, Uganda, Malawi, and Kenya found that opportunistic infections such as tuberculosis or cryptococcal meningitis were still the leading cause of death; severe bacterial infections were also prevalent and impacted survival, and almost a third of deaths were consistent with immune reconstitution inflammatory syndrome. Mortality rates were highest during the first 4 weeks on ART. In this trial, patients at highest mortality risk had a high burden of symptoms, weight loss, poor mobility, and low albumin and hemoglobin level [9]. The second study, from Kenya and DRC, reported that tuberculosis, malaria, cryptococcal meningitis, and pneumonia were the main causes of death among HIV-infected hospitalized patients. CD4 cell count remained the most significant determinant of mortality for all groups, regardless of gender, age, or type or number of comorbidities, and again mortality was highest in the early weeks after admission, with more than a quarter of deaths occurring within 48 hours of admission, suggesting that hospitalization occurred too late to prevent death in those severely sick patients [3]. Finally, a review article assessed risk factors for mortality in children with advanced HIV disease, highlighting that around 80% of children <5 years of age starting ART had advanced clinical and/or immunological staging [7]. The main causes for morbidity and mortality in children on ART are AIDS-related conditions (tuberculosis and pneumocystis pneumonia) and bacterial infections, followed by malnutrition and wasting, hematological disturbances, and in Africa, malaria [7].

What are the possible interventions that could reduce mortality from the main causes of mortality? A commentary summarizes recent guidance from the WHO for the management of advanced disease in a public health approach [10]. These guidelines recommend a targeted package of interventions to reduce mortality and morbidity from the major causes—tuberculosis, severe bacterial infections, and cryptococcal meningitis—as well as recommending rapid ART initiation and intensified adherence interventions. This package includes screening for cryptococcal antigenemia and tuberculosis, preemptive fluconazole treatment for those with cryptococcal antigenemia, and cotrimoxazole and isoniazid prophylaxis. Offering a package of care for patients in decentralized care, or hospitalized with advanced HIV disease, will help to focus attention on this group of patients with a high risk of death. It is anticipated that by differentiating ART service delivery and providing less frequent clinical visits for stable patients, clinic resources will be more equitably directed according to health needs.

The package of care still needs to be optimized. The commentary summarizes priorities for future research identified by the guideline development process and associated systematic reviews. In particular, antibiotic administration within a public health approach warrants further research to assess survival benefit against the potential risk for antibiotic resistance. Use of cryptococcal antigen (CrAg) screening is addressed by a systematic review that assessed CrAg positivity reported by 60 studies and suggests that raising the screening threshold to cell count ≤200 cells/µL to include all patients with advanced HIV disease may be beneficial in identifying an additional number of patients at risk of developing cryptococcal disease, and simplifying the approach to delivering the advanced HIV disease package by using a single threshold of 200 cells though further studies to quantify clinical benefit is required [11].

Overall, the aim of this supplement is to contribute to a renewed focus on responding to advanced HIV disease. Late HIV diagnosis and ART initiation increase dramatically the risk of dying from HIV and AIDS. Global goals aimed at reducing incidence and achieving epidemic control are welcome. At the same time, we must not lose sight of the continued need to reduce morbidity and mortality, which has always been and remains a central priority in the HIV response. ART provision on its own is not sufficient to reduce this mortality. An improved understanding of the epidemiology and opportunistic infection burden in patients with advanced HIV disease and the development of further evidence with respect to optimal screening, prophylaxis, diagnostic, and treatment approaches for these opportunistic infections are essential for informing practice and policy aimed at impacting mortality in patients with advanced HIV.

## Notes

**Supplement sponsorship.** This article appears as part of the supplement “Advanced HIV Disease,” sponsored by the World Health Organization.

***Potential conflicts of interest.*** A. C. has received unrestricted educational grants from Gilead, AbbVie, MSD, and ViiV Healthcare, and has received a research grant to the HIV Unit (Geneva University Hospitals) from Gilead Sciences SA. All other authors report no potential conflicts of interest. The authors have submitted the ICMJE Form for Disclosure of Potential Conflicts of Interest. Conflicts that the editors consider relevant to the content of the manuscript have been disclosed.
